# Unraveling the relative contribution of inter‐ and intrapopulation functional variability in wild populations of a tadpole species

**DOI:** 10.1002/ece3.3048

**Published:** 2017-05-23

**Authors:** Tian Zhao, Cheng Li, Xiaoyi Wang, Feng Xie, Jianping Jiang

**Affiliations:** ^1^ CAS Key Laboratory of Mountain Ecological Restoration and Bioresource Utilization and Ecological Restoration Biodiversity Conservation Key Laboratory of Sichuan ProvinceChengdu Institute of BiologyChinese Academy of SciencesChengduChina

**Keywords:** functional overlap, functional richness, functional traits, inter‐ and intrapopulation, intraspecific functional variability

## Abstract

Functional traits are increasingly recognized as an integrative approach by ecologists to quantify a key facet of biodiversity. And these traits are primarily expressed as species means in previous studies, based on the assumption that the effects of intraspecific variability can be overridden by interspecific variability when studying functional ecology at the community level. However, given that intraspecific variability could also have important effects on community dynamics and ecosystem functioning, empirical studies are needed to investigate the importance of intraspecific variability in functional traits. In this study, 256 *Scutiger boulengeri* tadpole individuals from four different populations are used to quantify the functional difference between populations within a species, and the relative contribution of inter‐ and intrapopulation variability in functional traits. Our results demonstrate that these four populations differ significantly in functional attributes (i.e., functional position, functional richness, and low functional overlap), indicating that individuals from different populations within a species should be explicitly accounted for in functional studies. We also find similar relative contribution of inter‐ (~56%) and intrapopulation (~44%) variation to the total variability between individuals, providing evidence that individuals within populations should also be incorporated in functional studies. Overall, our results support the recent claims that intraspecific variability cannot be ignored, as well as the general idea of “individual level” research in functional ecology.

## 1 INTRODUCTION

Investigating the relationship between biodiversity and ecosystem functioning is a central question in modern ecology (Cardinale et al., 2012; Loreau, 2001). Previous studies often focused on taxonomic diversity, despite the fact that biodiversity had a multitude of facets (Gaston, 1996; Purvis & Hector, 2000). In recent decades, however, an increasing number of literature is going beyond taxonomic diversity by focusing on functional attributes of communities (Albert et al., 2012). This is because both species identities and species functional traits (i.e., any biological attributes that can affect the fitness of organisms; Violle et al., 2007; Díaz et al., 2013) can affect the properties and processes of communities and ecosystems (e.g., predation: Rudolf, 2006, 2008; competition: Araújo et al., 2008; : food webs: Rudolf, Rasmussen, Dibble, & Van Allen, 2014; nutrient recycling and fluxes: El‐Sabaawi et al., 2015). To obtain functional traits in animal species, the ratios between morphological traits are typically used to estimate their vital functions performed in ecosystems (e.g., foraging movements in birds, Ricklefs, 2012; Dehling et al., 2014; food acquisition and locomotion in fish, Mason, Irz, Lanoiselée, Mouillot, & Argillier, 2008; Mason, Lanoiselée, Mouillot, Wilson, & Argillier, 2008; Villéger, Miranda, Hernández, & Mouillot, 2010; Albouy et al., 2011; food acquisition and habitat use in tadpoles, Altig & Johnston, 1989; Harris, 1999; Strauß, Reeve, Randrianiaina, Vences, & Glos, 2010).

Based on the implicit assumption that the effects of intraspecific variability (i.e., both inter‐ and intrapopulation functional variability within the same species) can be overridden by interspecific variability (i.e., functional variability among species) when studying functional ecology at the community level (McGill, Enquist, Weiher, & Westoby, 2006), conspecific individuals are primarily treated as ecologically equivalent. Therefore, mean species functional trait values are applied to describe the functional characteristics of organisms and calculate functional diversity indices (e.g., Schütz & Schulze, 2015; Villéger et al., 2010). However, a key tenet of functional ecology is that species are not equal, and individuals within a species or even within a population can differ in many biological and ecological traits such as fecundity, survival, or size (Bolnick et al., 2011; Vindenes, Engen, & Sæther, 2008). Particularly, ecological studies have widely indicated the differences of ecomorphological traits in conspecific individuals within the same species (Bolnick et al., 2003, 2011), which could be driven by differences in resource use (Skulason & Smith, 1995), ontogeny (Hjelm, Persson, & Christensen, 2000; Johansson, Rådman, & Andersson, 2006; Larson, 2005), or trophic specialization of individuals (Bolnick, Yang, Fordyce, Davis, & Svanbäck, 2002; Svanbäck & Bolnick, 2005).

Accordingly, high intraspecific variability in functional traits has been demonstrated by empirical studies in wild populations of plants (interpopulation; Jung, Violle, Mondy, Hoffmann, & Muller, 2010), invertebrates (intrapopulation but different stage structures; Rudolf & Rasmussen, 2013b), and fish (intrapopulation but different stage structures; Zhao, Villéger, Lek, & Cucherousset, 2014). More importantly, intraspecific variation in functional traits could have cascading effects on ecological processes (e.g., community assembly and dispersal; de Bello et al., 2011; Bestion, Clobert, & Cote, 2015), the calculation of functional diversity indices (Albert et al., 2012; Cianciaruso, Batalha, Gaston, & Petchey, 2009; Rudolf et al., 2014), and ecosystem functioning (e.g., total decomposition rates, net primary productivity, nutrient recycling and nutrient fluxes; Harmon et al., 2009; Rudolf & Rasmussen, 2013a; El‐Sabaawi et al., 2015). Therefore, quantifying the importance and the drivers of intraspecific variation in functional traits is of utmost importance to accurately calculate functional diversity indices and to better understand ecological patterns and processes (Bolnick et al., 2011; Violle et al., 2012).

A previous study found that different life stages of largemouth bass (*Micropterus salmoides*) occupied distinct functional niche space (i.e., different functional niche size and low functional overlap), primarily driven by ontogenetic shift and individual specialization (Zhao et al., 2014). It was also suggested that such low functional overlap could decrease the stability of ecological networks (Rudolf & Lafferty, 2011). However, there are still few empirical studies that have quantified the intraspecific functional trait variability and overlap in amphibian species. Furthermore, intraspecific variation can be studied at different scales, such as within and between populations (Mitchell & Bakker, 2014). Although much of the intraspecific variation can be explained by genetic differences (Begg, Wishart, Young, Squire, & Iannetta, 2012), intrapopulation variation can also reveal resource use and ontogeny (Zhao et al., 2014), while interpopulation variation can reflect the environmental adaptation of species (Kyle & Leishman, 2009). Therefore, studying the relative contribution of variation within and between populations can help ecologists to organize data collection, analysis, and interpretation (Mitchell & Bakker, 2014). In this study and using an anuran species larvae (i.e., tadpoles) as models, we quantified (1) the functional difference (i.e., functional position: the significance of the proximity, functional richness, and pairwise functional overlap) between four populations within a species and (2) to determine the relative contribution of inter‐ and intrapopulation variability in functional traits.

## 2 MATERIAL AND METHODS

### 2.1 Model species and specimen

*Scutiger boulengeri* is a widely distributed anuran species in high altitude areas of China, such as Tibet and western Sichuan province (Fei et al., 2009). Tadpoles of *Scutiger boulengeri* were selected as models as they have a long larval period before metamorphosis (i.e., approximate 5 years; Fei et al., 2009). And phenotypic plasticity has been observed in larval development rate of this species, with individuals altering development rate in response to changes in the environment (Fei et al., 2009). A total of 256 specimens (formaldehyde stored) preserved in the Herpetological Museum of Chengdu Institute of Biology, Chinese Academy of Sciences, were selected and measured. These individuals were from four different populations in southwest China, including 47 from Mangkang (98.60º N and 29.68º E), 60 from Basu (96.92º N and 28.37º E), 53 from Yadong (88.90º N and 27.48º E), and 96 from Kangding (101.97º N and 30.05º E). Individuals from different stages were pooled together as each individual can exhibit distinct functional traits within the population (Bolnick et al., 2003; Violle et al., 2012; Zhao et al., 2014).

### 2.2 Data acquisition

Each specimen was rinsed in distilled water and then measured for a set of 10 quantitative external morphological traits directly using Mshot Image Analysis System (Mc50‐N) on a stereomicroscope (JSZ8T, Jiang Nan Yong Xin, China) and a digital caliper to the nearest 0.1 mm in the laboratory. The development stages of tadpoles were determined according to Gosner (1960). All the measurements were conducted by the same person to ensure consistency.

### 2.3 Traits selection

**Figure 1:**
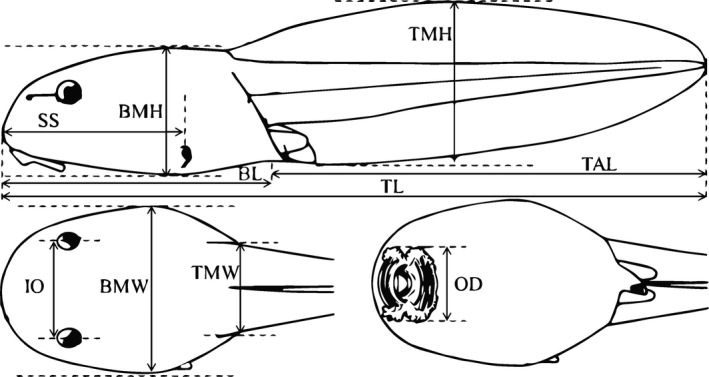
The measurement of 10 external morphological traits of tadpoles. Details of abbreviations are as follows: TMW, tail muscle width; TAL, tail length; BL, body length; TMH, tail muscle height; BMH, body maximum height; BMW, body maximum width; TL, total length; OD, oral disk width; SS, distance from tip of snout to opening of spiracle; IO, interocular distance (adapted from Haas & Das, 2011)

Based on the criteria that functional traits should be easily quantified on a large number of individuals (Dumay, Tari, Tomasini, & Mouillot, 2004), and on the basis of published literatures (Azizi, Landberg, & Wassersug, 2007; Eidietis, 2006; Grosjean, Randrianiaina, Strauß, & Vences, 2011; Grosjean, Strauß, et al., 2011; Raharivololoniaina, Grosjean, Raminosoa, Glaw, & Vences, 2006; Strauß et al., 2010; Van Buskirk & McCollum, 2000), nine complementary functional traits were selected to reflect the main ecological functions of tadpoles in freshwater ecosystems. These traits include total length (TL), body length (BL), body maximum height (BMH), body maximum width (BMW), tail length (TAL), tail muscle width (TMW), tail muscle height (TMH), oral disk width (OD), interocular distance (IO), and distance from tip of snout to opening of spiracle (SS; Figure 1; Glos, Teschke, and Vences (2007); Fei et al., 2009; Aguayo, Lavilla, Vera Candioti, & Camacho, 2009; Baldo, Maneyro, & Laufer, 2010; Grosjean, Strauß, et al., 2011). Importantly, as morphological changes across different development stages of tadpoles can be driven by individual size, all of these functional traits were unitless ratios that were a priori independent of individual body size (Winemiller, 1991; Villéger et al., 2010). Specifically, these functional traits described food acquisition (i.e., oral disk shape, oral disk position, eye position) and locomotion (i.e., tail shape, tail position, tail throttling, body section shape, trunk bending shape, spiracle position) in tadpoles (details in Table 1). For instance, oral disk shape provided information about the type of prey that tadpoles could capture in water bodies. Individuals with lower oral disk shape values tended to feed on small prey, while higher oral disk shape values indicating that the mouths of these individuals were large and round (Grosjean, Strauß, et al., 2011). Trunk bending shape represented the swimming type and endurance of tadpoles, with higher values indicating greater magnitude of vertebral curvature while lower values indicating some dorso‐ventral flexion, but little lateral flexion (Azizi et al., 2007).

**Table 1:** List of the nine functional traits associated with food acquisition and locomotion. The letter in brackets indicates the function associated with each trait (F, food acquisition and L, locomotion). Coefficients of variation (CV) were measured according to all the individuals

Functional traits	Measure	Ecological meaning	CV, %
Oral disk shape (F)	OD/BMW	Prey shape and food acquisition	9.7
Oral disk position (F)	OD/BL	Position of prey in the water	9.1
Eye position (F)	IO/BMW	Prey detection	15.4
Tail shape (L)	TMW/BMW	Hydrodynamism and Endurance,	15.5
Tail position (L)	TAL/BL	Endurance, acceleration, and/or maneuverability	10.6
Tail throttling (L)	TMH/BMH	Propulsion and/or maneuverability	12.6
Body section shape (L)	BMW/BMH	Position in the water column and hydrodynamism	10.5
Trunk bending shape (L)	BL/TL	Swimming type (magnitude of lateral bending of the trunk) and endurance	6.7
Spiracle position (L)	SS/BL	Swimming and hydrodynamism	7.6

TMW, tail muscle width; BW, body width; TAL, tail length; BL, body length; TMH, tail muscle height; BMH, body maximum height; BMW, body maximum width; TL, total length; OD, oral disk width; SS, distance from tip of snout to opening of spiracle; IO, interocular distance.

### 2.4 Statistical analyses

All the aforementioned functional traits were scaled to a mean of 0 and a standard deviation of 1 in order to give the same weight to each trait (Villéger, Mason, & Mouillot, 2008). To quantify the functional difference between populations, a principal component analysis (PCA) was first computed based on scaled functional trait values measured on all the individuals to build a multidimensional functional space. The first four synthetic principal components of the PCA (eigenvalues >1) were then selected as synthetic axes. We used permutational multivariate analysis (PERMANOVA, 9,999 permutations; Anderson, 2001) on the first four axes to test the significance of the proximity (i.e., functional position) between populations. Functional richness and functional overlap between populations were tested as follows: We first calculated convex hull areas in the functional space filled by four populations (i.e., observed functional richness) and the observed pairwise functional overlap between populations. A bootstrap procedure with 10,000 random subsets of 47, 53, 60 individuals, respectively (i.e., the minimum number of individuals within the four populations) from each population was then used to calculate the bootstrap functional richness and functional overlap. The comparison of functional richness difference between pairwise populations was based on the bootstrap results which were calculated using the maximum number of individuals, as increasing the number of bootstrap samples will always increase the accuracy of the test (Davidson & MacKinnon, 2000).

Due to known issues with calculating *R* ^2^ values from linear mixed models, we followed the method laid out by Nakagawa and Schielzeth (2013) for computing the relative contribution of inter‐ and intrapopulation variation for each functional trait. Specifically, we constructed a full GLMMs model [i.e., functional trait ~ Population + (1|Stage)] that included population as the fixed effects and a random intercept stage effect. Marginal *R* ^2^ (i.e., proportion of variance explained by the fixed effects) was calculated as:

$$R_{m}^{2}=\frac{\sigma_{f}^{2}}{\sigma_{f}^{2}+\sigma_{r}^{2}+\sigma_{\epsilon }^{2}}$$

while conditional *R* ^2^ (i.e., proportion of variance explained by both the fixed and random effects) can be expressed as:

$$R_{c}^{2}=\frac{\sigma_{f}^{2}+\sigma_{r}^{2}}{\sigma_{f}^{2}+\sigma_{r}^{2}+\sigma_{\epsilon }^{2}},$$

where $R_{m}^{2}$ was the proportion of interpopulation variation, $R_{c}^{2}$ was the proportion of both inter‐ and intrapopulation variation, $\sigma_{f}^{2}$ was the variation calculated from the fixed effects, $\sigma_{r}^{2}$ was the variation component of random effects, and $\sigma_{\epsilon }^{2}$ was the residual variation. Proportion of intrapopulation variation can be identified as the difference between $R_{m}^{2}$ and $R_{c}^{2}$. All statistical analyses were conducted in R 3.2.2 (R development Core Team 2011).

## 3 RESULTS

The tadpole specimens were from stage 26 to stage 41 (Appendix S1), and the total length ranged from 32.4 to 77.4 mm (mean = 56.0 ± 8.2 *SD*). Each of the nine functional traits had very high total intraspecific variability, with the mean coefficient variation = 10.9% ± 3.1% *SD* (Table 1). Combined the first four axis explained 81.86% of the total inertia (PC1 = 35.70%, PC2 = 18.51%, PC3 = 15.35%, PC4 = 12.30%, respectively; Table 2). Specifically, PC1 was principally correlated with functional traits related to both food acquisition and locomotion. Therefore, individuals could display larger and rounded mouth, more propulsion and/or maneuverability but lower endurance (dorso‐ventral flexion) along the increasing of PC1 values. PC2 was principally driven by functional traits related to locomotion, indicated that with increased PC2 values, individuals were more compact and rounded, but less propulsion and/or maneuverability (i.e., lower movement precision; Wassersug, 1989; Hoff & Wassersug, 2000; Van Buskirk & McCollum, 2000; Larson & Reilly, 2003; Mcnamara et al., 2009; Aguayo et al., 2009; Johnson, Saenz, Adams, & Hibbitts, 2015).

**Table 2:** Pearson correlation coefficients between the four principal components analysis axes and the nine functional traits. Significant *p*‐values are in bold

Functional traits	PC1 (35.70%)	PC2 (18.51%)	PC3 (15.35%)	PC4 (12.30%)
Oral disk shape	**0.62**	**−0.63**	**0.34**	**−0.24**
Oral disk position	**0.36**	**−**0.08	**−**0.08	**0.84**
Eye position	**0.43**	**0.28**	**−0.65**	**−0.41**
Tail shape	**0.26**	**−0.51**	**−**0.12	0.11
Tail position	**0.97**	**−0.13**	0.03	**−**0.03
Tail throttling	**0.45**	**0.52**	**0.31**	**−0.28**
Body section shape	**0.19**	**0.52**	**0.76**	0.11
Trunk bending shape	**−0.97**	0.12	−0.03	0.03
Spiracle position	**0.56**	**0.59**	**−0.39**	**0.27**

**Figure 2:**
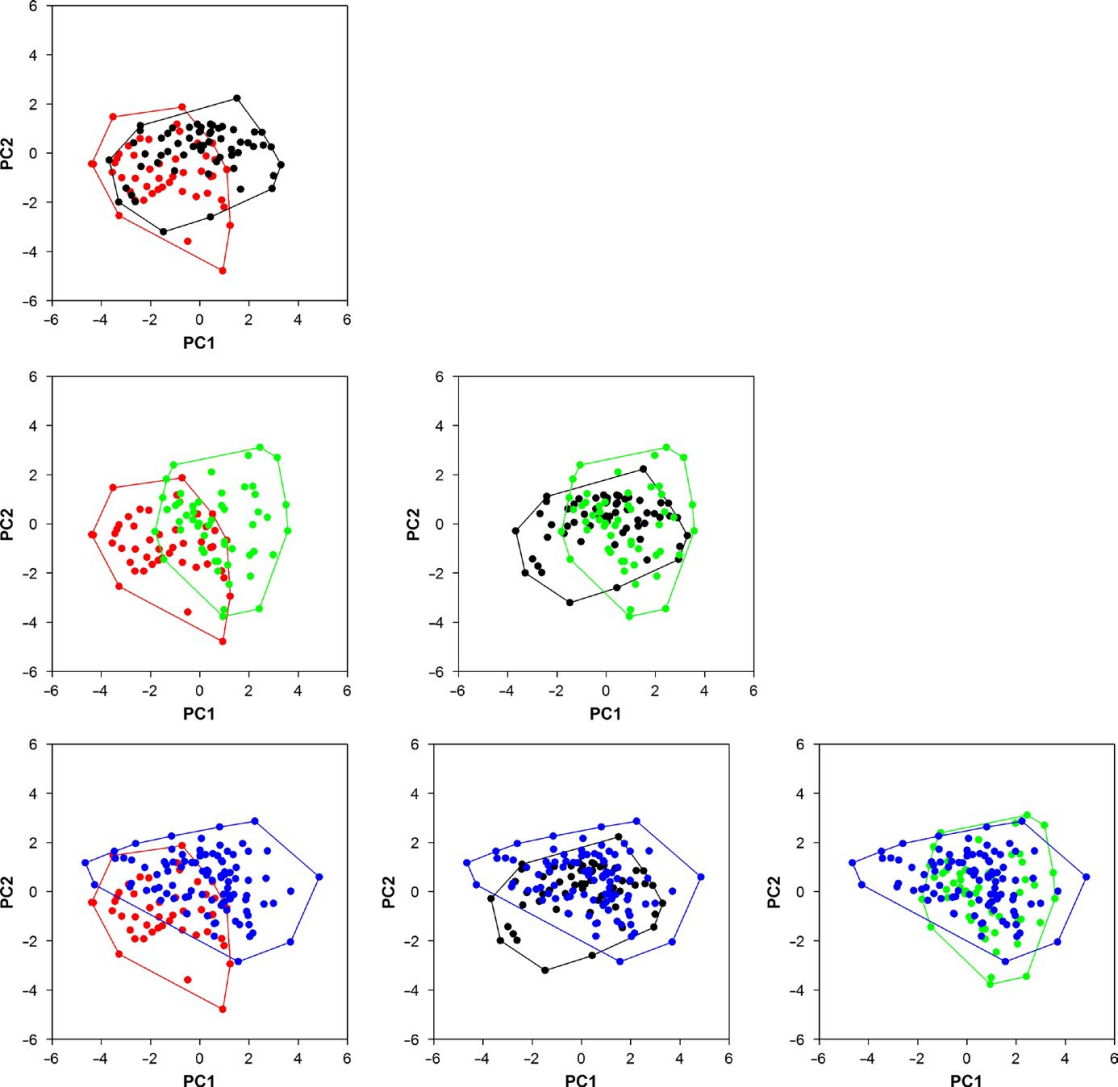
Distribution of *Scutiger boulengeri* tadpole individuals in the functional space (only the two‐first axes are shown). Individuals in Mangkang, Basu, Yadong, and Kangding populations are plotted in red, blue, green, and black, respectively. Functional richness is illustrated by the convex hull area with corresponding colored border

The position of individuals in the functional space significantly differed between four populations (PERMANOVA, *p *<* *.001, Figure 2). Observed functional richness was 13.24% for Mangkang (*n* = 47), 30.61% for Yadong (*n* = 53), 14.06% for Basu (*n* = 60), and 35.55% for Kangding (*n* = 96), respectively. Bootstrap test indicated that when considering 47 individuals, functional richness of Yadong and Kangding was significantly higher than that of Mangkang. However, there was no significant difference in functional richness between Basu and Mangkang (Table 3). When considering 53 individuals, functional richness of Basu and Kangding was significantly lower than that of Yadong (Table 3). In addition, when considering 60 individuals, functional richness of Kangding was significantly higher than that of Basu (Table 3). Observed pairwise functional overlap was 28.40% between Mangkang and Basu, 6.89% between Mangkang and Yadong, 6.14% between Mangkang and Kangding, 26.84% between Basu and Yadong, 21.33% between Basu and Kangding, and 32.82% between Yadong and Kangding. The pairwise functional overlap between populations was similar when considering 47 individuals from bootstrap test, with mean = 26.05% ± 2.48% *SD* between Mangkang and Basu, mean = 6.77% ± 0.80% *SD* between Mangkang and Yadong, mean = 6.65% ± 1.15% *SD* between Mangkang and Kangding, mean = 23.97% ± 3.12% *SD* between Basu and Yadong, mean = 23.75% ± 3.76% *SD* between Basu and Kangding, and mean = 27.18% ± 3.90% *SD* between Yadong and Kangding. When 15 individuals (i.e., the number of individuals that is usually used to calculate the mean functional trait values in animal species; e.g., Mason, Irz, et al., 2008; Villéger et al., 2010) were subsampled from each population (i.e., 31.9% of Mangkang population, 28.3% of Yadong population, 25.0% of Basu population, and 12.6% of Kangding population, respectively), the estimation of average functional richness corresponded to only 2.9% ± 1.1% *SD*, 5.9% ± 2.5% *SD*, 2.5% ± 1.0% *SD*, and 4.1% ± 1.6% *SD* of the observed functional richness of each population, respectively.

**Table 3:** Number of individuals from each population, observed, and bootstrapped functional richness considering 47, 53, or 60 individuals (95% confidence interval) of the four populations

Population	*n*	Functional richness			
		Observed	Bootstrapped_n=47_	Bootstrapped_n=53_	Bootstrapped_n=60_
Mangkang	47	13.24%	–	–	–
Yadong	53	30.61%	22.58%–30.40%	–	–
Basu	60	14.06%	8.60%–13.31%	10.50%–13.94%	–
Kangding	96	35.55%	13.28%–25.69%	15.26%–27.76%	17.71%–30.64%

**Figure 3:**
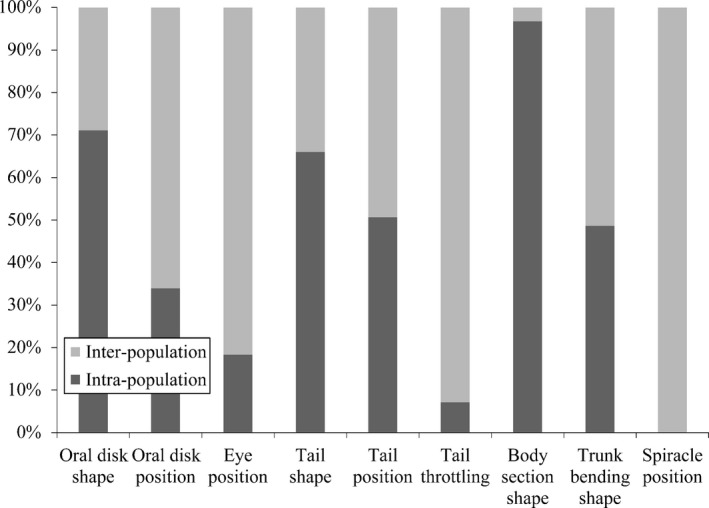
The decomposition of inter‐ and intrapopulation variation in nine functional traits. The light bars represent the relative contribution of interpopulation variation in each trait, while the dark bars are the relative contribution of intrapopulation traits variation

From the GLMMs model, $R_{m}^{2}$ values ranged from 1.38% to 25.61% (mean = 11.75% ± 7.30% *SD*) and $R_{c}^{2}$ values ranged from 6.32% to 42.43% (mean = 23.86% ± 10.14% *SD*; Table 4). When considering only the stages and the populations effects (i.e., without residual variance), the relative contribution of inter‐ and intrapopulation variation to the total variability between individuals was similar. Specifically, functional variation within populations explained an average of 43.63% ± 30.04% *SD* of the total variability, while functional variation between populations explained an average of mean = 56.37% ± 30.04% *SD* of the total variability. However, the partition between inter‐ and intrapopulation variability was strongly different in each functional trait. For instance, variation of spiracle position was totally explained by interpopulation traits variability (i.e., 100.00%, Figure 3), while body section shape showed the lowest interpopulation traits variability (i.e., 3.25%, Figure 3) among the nine functional traits.

**Table 4:** Results of GLMMs models used to test the fixed effects variation (i.e., $\sigma_{f}^{2}$), random‐effects variation (i.e., $\sigma_{r}^{2}$), residual variation (i.e., $\sigma_{\in }^{2}$), marginal *R* ^*2*^ (i.e., $R_{m}^{2}$), and conditional *R* ^*2*^ (i.e., $R_{c}^{2}$) values

Functional traits	$\sigma_{f}^{2}$	$\sigma_{r}^{2}$	$\sigma_{\in }^{2}$	$R_{m}^{2}$	$R_{c}^{2}$
Oral disk shape	0.0046	0.0113	0.0595	6.06%	21.00%
Oral disk position	0.0000	0.0000	0.0006	4.18%	6.32%
Eye position	0.0016	0.0004	0.0044	25.61%	31.36%
Tail shape	0.0003	0.0006	0.0029	7.65%	22.53%
Tail position	0.0049	0.0050	0.0233	14.72%	29.82%
Tail throttling	0.0022	0.0002	0.0157	11.99%	12.91%
Body section shape	0.0004	0.0116	0.0162	1.38%	42.43%
Trunk bending shape	0.0001	0.0001	0.0005	15.02%	29.23%
Spiracle position	0.0004	0.0000	0.0017	19.13%	19.13%

## 4 DISCUSSION

Our results demonstrated the high intraspecific variability in tadpole functional traits, primarily driven by both inter‐ and intrapopulation variation. This was because the traits variability explained by tadpole stages and populations was similar, suggesting that both stages and populations effects are important to affect functional trait variability within species. However, the partition between inter‐ and intrapopulation variability was strongly different for each functional trait.

Overall, the four *Scutiger boulengeri* populations were significantly different in the occupation of functional space. Specifically, both Mangkang and Basu populations had significant smaller functional richness than that of Yadong and Kangding populations. Despite more individuals were considered in Kangding population (*n* = 96), it had smaller functional richness than Yadong population (*n* = 53). Additionally, the patterns of functional position between the four populations were significantly different, which could be due to the low pairwise functional overlap. All of these observations indicated that these four populations displayed distinct functional properties (i.e., both position and richness) in the functional space. However, the magnitudes of traits variation between populations were strongly related to the environmental gradients of habitats (Albert et al., 2010). For instance, Pires, McBride, and Reznick (2007) found that two *Poeciliopsis prolifica* populations from the similar habitats did not differ significantly in life‐history traits. In contrast, Tamate and Maekawa (2000) demonstrated that *Oncorhynchus masou* populations in a low‐growth environment can exhibit some specific reproductive traits such as larger eggs and lower fecundity. In the present study, tadpoles from Mangkang population were the most functionally different individuals compare to others, which had higher trunk bending shape values, lower eye position values, and lower spiracle position values. These functional traits were more related to locomotion, indicating that the locomotion of these individuals was small magnitude of vertebral curvature but more endurance (i.e., some dorso‐ventral flexion, but little lateral flexion; Eidietis, 2006; Azizi et al., 2007). This probably because these individuals were sampled from the water bodies of Jinsha and Lancang rivers sutures that can have relative higher flow velocity. Given that framework and the potential environmental gradients among sampling sites, we guess that individuals from different populations probably exhibited phenotypic plasticity in response to environmental changes and then improve their performance within the ecosystems (Relyea, 2001; Relyea & Werner, 2000; Van Buskirk, 2002). However, as only functional trait variability was identified in the present study, additional studies that combined traits variation with environmental gradients were needed to confirm our conclusions. In addition, our results were also consistent with previous studies showing that traditional method which randomly selected several individuals from only one population could disproportionally affect the investigation of the functional properties within a species, or estimation functional diversity of communities (Mitchell & Bakker, 2014; Zhao et al., 2014). More importantly, such interpopulation functional variability likely influenced the functions that tadpoles played within communities, suggesting that without account for it may bias estimates of ecosystem functioning (Mitchell & Bakker, 2014; Post, 2003).

The variation within populations was due to functional traits related to both food acquisition (e.g., oral disk shape) and locomotion (e.g., body section shape and tail shape). It is widely observed in animal species that ontogenetic shift and individual specialization can induce the change of traits, probably associated with diet shift, foraging behavior modification, and the mobility of prey encountered (Zhao et al., 2014). For instance, the fish largemouth bass individuals can display deeper body, thicker caudal peduncles, and more rounded pectoral fins from consuming zooplankton, macroinvertebrates to fish (Post, 2003). For tadpoles species like *Hyla chrysoscelis*, individual in stage 20 can have a roughly semicircular mouth with the convex side anterior (i.e., mainly feed on attached material from submerged substrates), while individual in stage 24 usually has a C‐shaped oral pad (i.e., consume occasional zooplanktons and remove some fragile periphyton from substrates; Thibaudeau & Altig, 1988). Therefore, the relative contribution of variation within populations in the present study could be due to the different food acquisition and habitat use of *Scutiger boulengeri* tadpole individuals. However, much more evidence should be provided to understand how stage structures and individual specialization within a tadpole species can drive the intrapopulation traits variation in the future studies.

Overall, our studies supported the claims that intraspecific traits variability cannot be ignored in functional ecology (Violle et al., 2012). The distinct functional space occupation of four *Scutiger boulengeri* populations suggested that individuals from different populations within a species should be explicitly accounted for in functional studies. This was especially true in populations from large environmental gradients, because individuals from these populations usually possessed a diverse of functional traits, allowing them to persist through particular environmental conditions, thereby stabilizing function (Bolnick et al., 2011; Hooper et al., 2005). Similar relative contribution of intrapopulation variation to the total variability between all the individuals suggested that variation within populations should also be incorporated in functional studies, because such variation can greatly change population dynamics, trophic structures, and ecosystem functioning (Bolnick et al., 2003). Despite theory has been provided, more empirical studies were needed to exploit the ecological consequences of both inter‐ and intrapopulation functional variability.

## CONFLICT OF INTEREST

None declared.
