# Supplementary material for: Unraveling the relative contribution of inter‐ and intrapopulation functional variability in wild populations of a tadpole species
Source: Ecol Evol. 2017 May 23;7(13):4726–34. doi: 10.1002/ece3.3048 (PMC5496530; doi:10.1002/ece3.3048)
Supplement: Supplementary file 1 [file ECE3-7-4726-s001.docx]

**Appendix A** The number of individuals of each development stage from different populations.

| **Stages** | **Populations** | | | |
| --- | --- | --- | --- | --- |
|  | **Mangkang** | **Basu** | **Yadong** | **Kangding** |
| 26 | 0 | 0 | 0 | 3 |
| 27 | 0 | 0 | 1 | 1 |
| 28 | 0 | 0 | 0 | 1 |
| 29 | 0 | 0 | 2 | 0 |
| 30 | 0 | 0 | 0 | 2 |
| 31 | 0 | 0 | 1 | 4 |
| 32 | 0 | 4 | 3 | 3 |
| 33 | 0 | 6 | 3 | 2 |
| 34 | 0 | 1 | 1 | 12 |
| 35 | 12 | 6 | 9 | 17 |
| 36 | 9 | 15 | 20 | 36 |
| 37 | 11 | 8 | 5 | 9 |
| 38 | 12 | 14 | 7 | 3 |
| 39 | 0 | 0 | 0 | 0 |
| 40 | 3 | 6 | 1 | 2 |
| 41 | 0 | 0 | 0 | 1 |
